# Genomic diversity of *Taylorella equigenitalis* introduced into the United States from 1978 to 2012

**DOI:** 10.1371/journal.pone.0194253

**Published:** 2018-03-27

**Authors:** Jessica Hicks, Tod Stuber, Kristina Lantz, Matthew Erdman, Suelee Robbe-Austerman, Xiaoqiu Huang

**Affiliations:** 1 Diagnostic Bacteriology and Pathology Laboratory, National Veterinary Services Laboratories (NVSL), USDA, Ames, IA, United States of America; 2 Policy Evaluation and Licensing Section, Center for Veterinary Biologics, USDA, Ames, IA, United States of America; 3 Department of Computer Science, Iowa State University, Ames, Iowa, United States of America; Oklahoma State University, UNITED STATES

## Abstract

Contagious equine metritis is a disease of worldwide concern in equids. The United States is considered to be free of the disease although sporadic outbreaks have occurred over the last few decades that were thought to be associated with the importation of horses. The objective of this study was to create finished, reference quality genomes that characterize the diversity of *Taylorella equigenitalis* isolates introduced into the USA, and identify their differences. Five isolates of *T*. *equigenitalis* associated with introductions into the USA from unique sources were sequenced using both short and long read chemistries allowing for complete assembly and annotation. These sequences were compared to previously published genomes as well as the short read sequences of the 200 isolates in the National Veterinary Services Laboratories’ diagnostic repository to identify unique regions and genes, potential virulence factors, and characterize diversity. The 5 genomes varied in size by up to 100,000 base pairs, but averaged 1.68 megabases. The majority of that diversity in size can be explained by repeat regions and 4 main regions of difference, which ranged in size from 15,000 to 45,000 base pairs. The first region of difference contained mostly hypothetical proteins, the second contained the CRISPR, the third contained primarily hemagglutinin proteins, and the fourth contained primarily segments of a type IV secretion system. As expected and previously reported, little evidence of recombination was found within these genomes. Several additional areas of interest were also observed including a mechanism for streptomycin resistance and other virulence factors. A SNP distance comparison of the *T*. *equigenitalis* isolates and *Mycobacterium tuberculosis complex* (MTBC) showed that relatively, *T*. *equigenitalis* was a more diverse species than the entirety of MTBC.

## Introduction

Contagious equine metritis (CEM) is a venereal disease of equids caused by the bacteria *Taylorella equigenitalis*. This gram-negative coccobacillus is a member of the *Alcaligenaceae* family and is one of two species in the genus. It is only known to infect equids, and colonization in males is asymptomatic with bacteria co-existing among normal skin flora. Infection in females is often characterized by copious vaginal discharge and acute infertility, however, symptoms range from severe to undetectable [1–4].

This disease is currently present or suspected in 9 countries throughout the world, according to OIE, and another 39 have had incidental outbreaks of the disease, including the United States. The CEM disease status of many countries is unknown due to the absence of monitoring and import programs. This makes the diagnosis important in not only clinical cases but also animals with sub-clinical infection [5]. Missed diagnoses of animals, especially those that are asymptomatic, leaves countries vulnerable to new introductions. These introductions pose a great risk for countries where the organism is not endemic, like the United States, and are key to the insidious movement of the organism throughout the world [3, 4, 6].

Employing molecular typing methods to study the epidemiology of cases of CEM has been in practice for over 25 years. Several molecular typing studies using gel electrophoresis have been published in an effort to characterize and classify *T*. *equigenitalis* for epidemiological tracing. These studies have often showed little or no variation between isolates. The first restriction enzyme/gel-electrophoresis study of this organism published in 1990 showed 5 groups across 32 isolates [7]. In 1994, chromosomal DNA fingerprinting revealed only a small degree of difference among 28 isolates with 18 being identical, leading the authors to conclude there is high degree of genetic homogeneity among *T*. *equigenitalis* isolates [8]. Further research showed that in all 109 Japanese isolates between 2 studies there was a single, identical electrophoresis pattern with pulsed-field gel-electrophoresis (PFGE), but these differed from strains originating in other parts of the world [9, 10]. Despite these large groupings, PFGE provided the most distinguishing genetic characteristics among isolates for many years with studies showing as many as 17 groups in 82 isolates [6]. Other attempted methods, random amplified polymorphic DNA and amplified rDNA restriction analysis, made previously distinguishable isolates appear identical [11].

Whole genome sequencing of *T*. *equigenitalis* and *Taylorella asinigenitalis* published in 2012 was the first look at the genomics of the genus and the potential molecular diversity [12, 13]. Comparison of the two species showed some distinct characteristics, but also a large degree of similarity [12]. The comparison of 3 isolates of *T*. *equigenitalis* identified 3 primary regions of the genome containing significant differences between isolates, while also showing much of the genome is homologous [13]. The whole genome sequencing information lead to the development of a multi-locus sequence typing scheme (MLST) for the genus *Taylorella* [14]. This method expanded the distinguished groups to 27 in just the 113 isolates evaluated in the study.

While MLST is by far the most discerning typing method currently available, a single sequence type can be observed across epidemiologically unrelated outbreaks (13). Further characterization of the molecular diversity is necessary for the development of higher resolution techniques to better understand the epidemiology of the species. The need for such characterization is especially evident during disease outbreaks when determining source, direction of transmission, and association to other isolates would be very helpful for containing and eradicating the disease.

In order to gain additional genetic insight and advance the genetic typing methods for this species, a large molecular comparison study based on whole genome sequencing that spans both multiple geographical regions and time is necessary. The National Veterinary Services Laboratories’ (NVSL) collection of diagnostic isolates of *T*. *equigenitalis* dates back to 1978 and includes samples introduced into the United States during horse importation from various countries around the world. In this study, our objectives were to create quality reference genomes that represent the genetic diversity of these isolates and to achieve a better understanding of this organism from a molecular perspective. We used long and short read sequencing technologies to create these genomes to allow for the most accurate and complete assembly with the current available technology. Here we describe differences between these genomes compared to previously published references, as well as other areas of interest.

## Materials and methods

### Isolate diversity

To ensure diverse isolates were selected for the study, all 200 isolates of *T*. *equigenitalis* from the NVSL’s isolate repository were initially sequenced using an Illumina MiSeq, de novo assembled and then analyzed using kSNP (methods described below). Five diverse isolates were then selected for this study (Table 1) which represented each major branch in this tree. Within clade selection of each isolate was guided using PFGE results to ensure no duplicate patterns were included. (S1 Fig) Once assembly was complete, the MLST of each isolate was determined using the *Taylorella* MLST Database [14–16]. *In silico* PFGEs were also performed at the *ApaI* restriction site (5’-GGGCC|C-3’).

**Table 1:** *Taylorella equigenitalis* isolate information.

Isolate	GenBank Accession	Collection Year	Animal Country of Origin	Breed	Gender	Epidemiology Summary
79–1587	CP021060	1978	United States	Thoroughbred	Female	Bred to a European stallion
89–0490	CP021199	1989	Germany	Unknown	Female	No Data
92–0972	CP021200	1992	Austria	Lipizzaner	Male	No Data
98–0554	CP021201	1998	Netherlands	Unknown	Female	No Data
09–0932	CP021246	2009	United States	Quarter Horse	Female	2009 US outbreak of unknown origin [4]

Summary of isolate information showing relevant collection data and GenBank Accession numbers.

### Culture, extraction & sequencing

Isolates were grown on Eugon agar from frozen culture. After 48–72 hours of incubation at 37°C in 5% CO_2_, colonies were selected and plated on to fresh Eugon agar to ensure pure culture for extraction. DNA was extracted (Epicentre Masterpure kit, Epicentre, Madison, WI) at 48–72 hours postinoculation and sequenced using NexteraXT library prep (Illumina, San Diego, CA) on a Miseq (Illumina). For long read sequencing DNA was extracted (DNeasy Blood and Tissue kit, Qiagen, Hilden, Germany) libraries prepared with 15kb-20kb insert size (BluePippin kit) and sequenced on an RS II (Pacific Biosciences, Menlo Park, CA) with 2 SMRT cells per sample.

### Assembly & alignment

Short-read sequences were *de novo* assembled using ABySS [17] for kSNP analysis. PacBio reads were assembled using HGAP version 2.0 with the default parameters [18]. Canu version 1.4 was also used to assemble raw PacBio reads [19]. Mauve was used to align the output of both HGAP and Canu for comparison [20]. Burrows-Wheeler Aligner (BWA)-MEM algorithm was used to align Illumina MiSeq short-reads from the same isolate to the HGAP and Canu outputs separately [21]. The alignment data was used to resolve the differences between the HGAP and Canu assemblies, and the short-read data from the Illumina MiSeq was once again aligned to the final long-read assembly with BWA-MEM algorithm to ensure that the proper alignment of the short-reads was maintained. Integrated Genome Viewer (IGV) was used to visualize this alignment data [22].

The final assemblies of the 5 isolates were submitted to NCBI for annotation with the Prokaryotic Genome Annotation Pipeline (PGAP) [23]. Once completed, annotated genomes had been achieved, the 5 isolates along with NC_018108.1 and NC_014914.1 were aligned using Mauve. BLAST Ring Image Generator (BRIG) was used to create a circular genome comparison to highlight the areas of difference and similarity between the five genomes compared to each reference [24]. Geneious version 9.1.8 was used to manage and compile the different data types [25, 26].

Quantification of the diversity observed from the Mauve and BRIG alignments was achieved by aligning the MiSeq reads of all 200 isolates of *T*. *equigenitalis* from the NVSL diagnostic repository to each of the five new assemblies with BWA-MEM as previously described. Samtools was used to output depth of coverage information for each sequence, and GATK’s UnifiedGenotyper was used to call single nucleotide polymorphisms (SNPs) [27, 28]. In-house developed scripts were used to calculate SNP rates and percent coverage at a minimum depth of 10X in 35,000 base pair contiguous windows across each genome. Mean and variance were then calculated for SNP rate and percent coverage for each window and compared. Overall percent identity and percent coverage for each pair of the study isolates was calculated from pairwise alignments in Lastz. [29]

### Data availability

There are two complete, finished genomes of *T*. *equigenitalis* publicly available, one of which is type strain ATCC 35865 (NC_018108). Both were included in this study (NC_018108.1 and NC_014914.1). Published data on an additional draft sequence was also used (NC_021036.1). The published *T*. *asinigenitalis* isolate was also used (NC_016043.1).

All sequence data, both assembled and raw, are available from NCBI in the nucleotide and Short Read Archive (SRA) databases under BioProjects 384636 and 385665. (S2 Table)

### Phylogenetic tree

A phylogenetic tree was constructed from the NVSL short-read assemblies using kSNP [30]. The parameter k was selected using the module kChooser. The resulting phylogenetic tree was then used to select representative isolates along the 5 major branches for sequencing with PacBio technology. A second kSNP tree was created with final assemblies of the 5 study isolates and all available *Taylorella* sp. isolates in GenBank. (S2 Fig)

## Results

### Isolate diversity

The 5 isolates investigated in this study represented much of the diversity in the NVSL collection of isolates. Not only did they represent each of the 5 major branches in the phylogenetic tree, but each isolate was also collected at a different point in time and represented a different MLST and PFGE pattern. (S1 Fig) The isolate attributes are listed in Tables 1 and 2.

**Table 2:** *Taylorella equigenitalis* isolate attributes.

Isolate	Length	GC	Streptomycin	PFGE	MLST (ST-CC)	Total Genes	Coding Genes
79–1587	1,739,054	37.3%	Resistant	TE003	1–1	1621	1546
89–0490	1,649,945	37.6%	Susceptible	TE018	16–2	1527	1445
92–0972	1,666,291	37.5%	Susceptible	TE011	4 –(no cc)	1534	1455
98–0554	1,692,042	37.5%	Resistant	TE014	17–4	1565	1486
09–0932	1,635,330	37.6%	Resistant	TE004	58 - (no cc)	1508	1434
NC_018108.1	1,732,123	37.3%	Resistant	TE003	1–1	1619	1536
NC_014914.1	1,695,860	37.4%	Unknown	Unknown	2–1	1577	1512

Summary of isolate assembly and typing. Shown are length of the completed sequence, GC content, Streptomycin resistance status, PFGE pattern (by *in vitro* analysis), and MLST (sequence type–clonal complex). [15]

### Assembly

Using both the PacBio and Illumina sequence data, all 5 genomes were successfully assembled to 1 contig. The average depth of coverage for the PacBio reads assembled in HGAP was 645X. The average depth of coverage from the alignment of the Illumina short reads to the final long read assembly was 198X. (S2 Table) The long-read assemblies using both HGAP and Canu were highly similar in all 5 isolates. Only isolate 89–0490 had a small second contig in the HGAP assembly, this contig did not exist in the Canu assembly. Short-read alignment data showed it to be a poorly covered assembly of non-unique reads, and it was removed from further analysis. All other long-read assemblies were a single contig. Assemblies were all of consistent and expected length and GC content compared with other *T*. *equigenitalis* isolates. *In silico* PFGEs at the *ApaI* restriction site (5’-GGGCC|C-3’) yielded fragment sizes consistent with those in the patterns resolved by traditional *in vitro* PFGE analysis of these isolates.

A comparison of these five new isolates along with previously published GenBank sequences of both *Taylorella* species using a phylogenetic tree was performed to better understand the genomic relationship between isolates. (S2 Fig)

### Alignments

**Fig 1:**
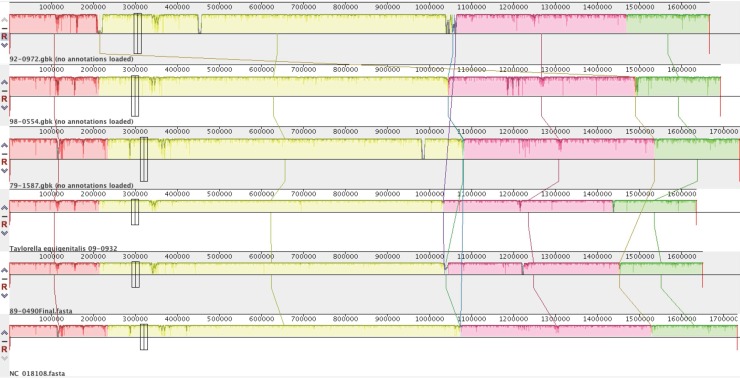
Mauve alignment. This alignment contains the 5 study isolates and NC_018108.1. Overall similarity among the 6 genomes with localized regions of variability is visible. No rearrangements are evident.

**Fig 2:**
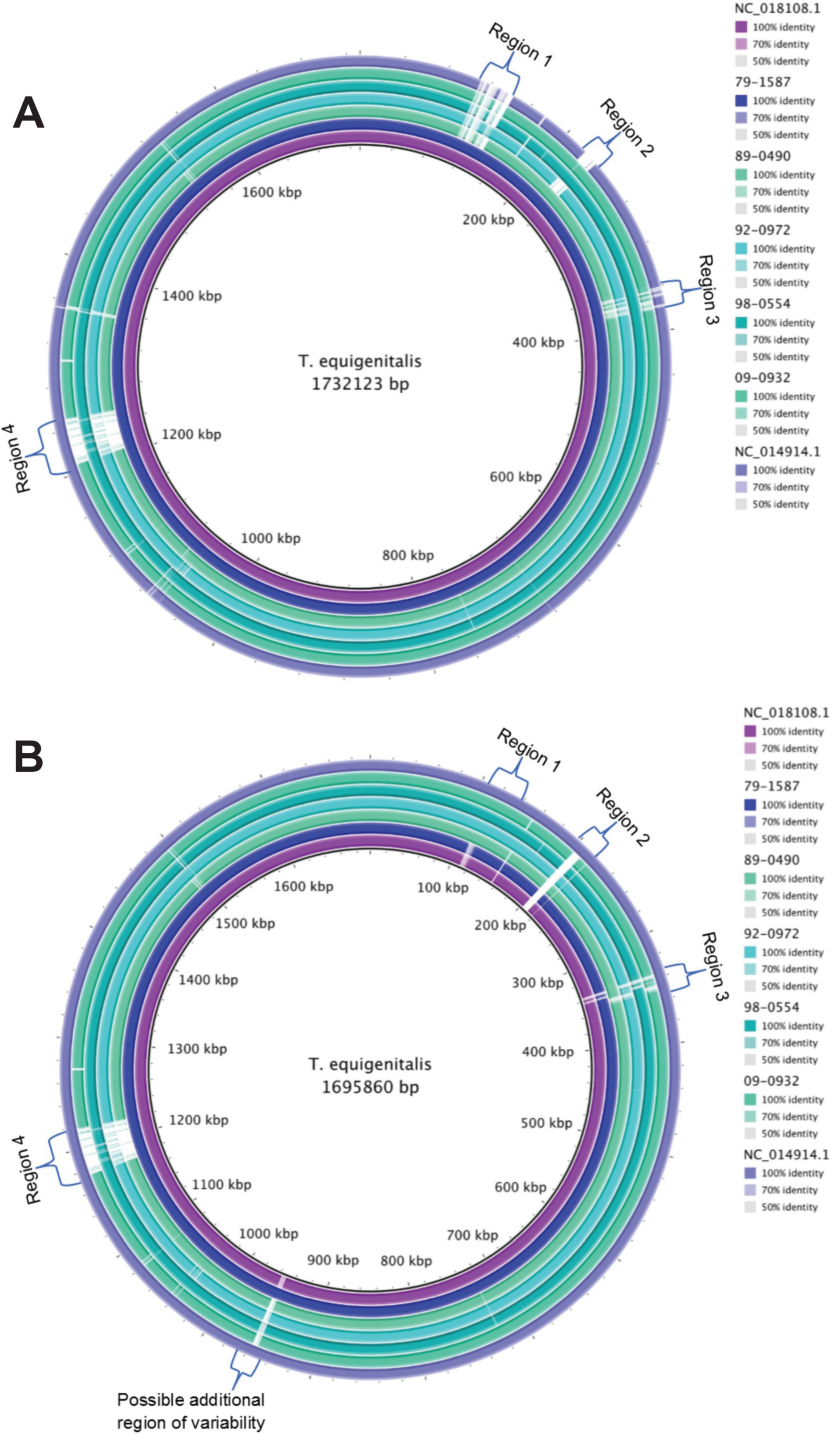
BRIG alignment. BRIG alignments of the 5 study isolates and the references. (A) Relative to reference NC_018108.1 four major regions with poor alignment are visible. (B) A possible fifth region with poor alignment becomes visible when isolates are aligned with respect to NC_014914.1.

**Fig 3:**
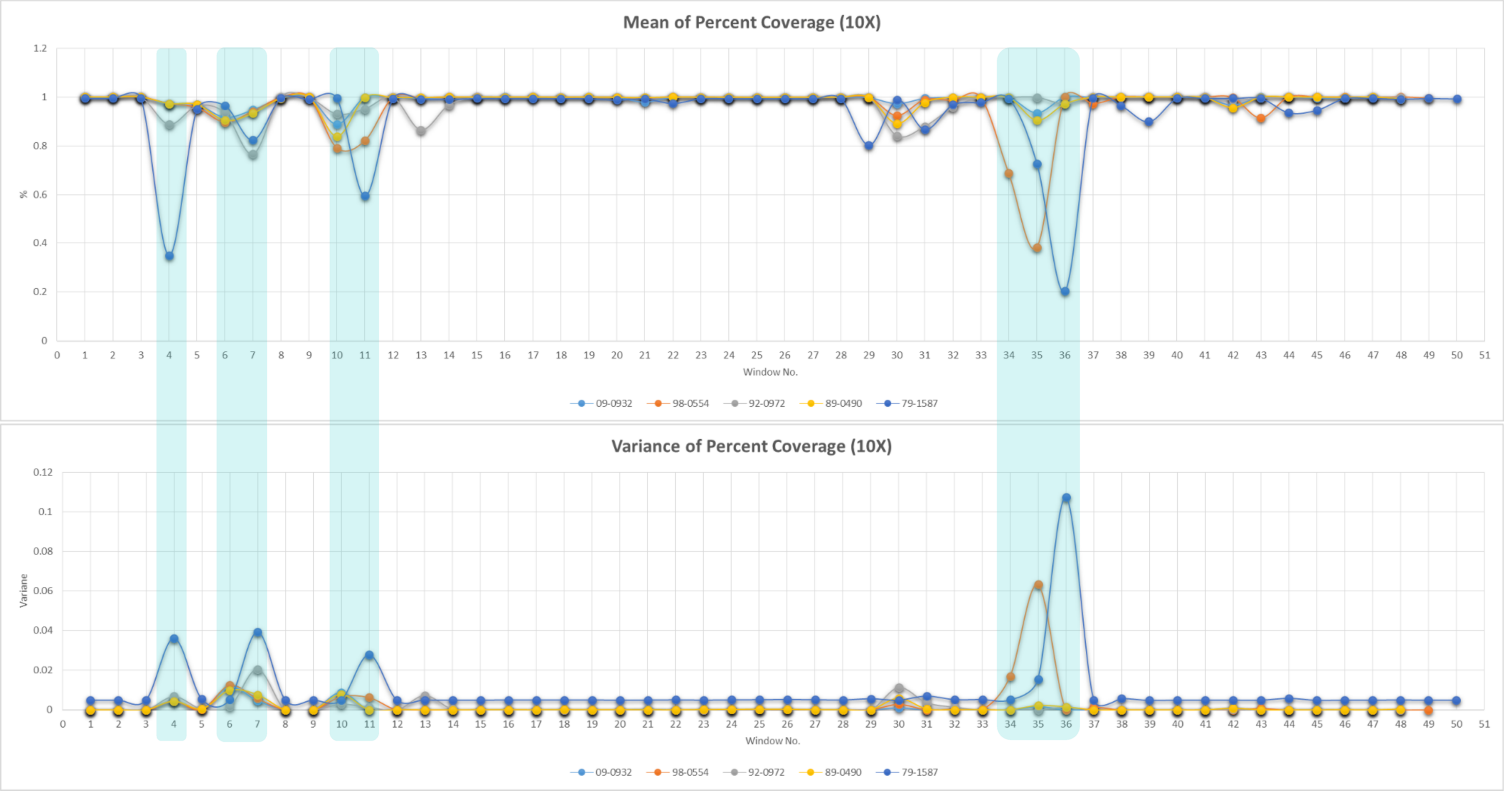
Mean and variance of percent coverage by window. Mean percent coverage and variance of the percent coverage of each isolate at a minimum depth of 10X using the MiSeq reads from the 200 NVSL diagnostic isolates of *T*. *equigenitalis*. Results are given by 35,000 bp window.

No major rearrangements or inversions were found from the alignment in Mauve. (Fig 1) The isolates are largely homologous with local variation. The BRIG alignment made it clear there were four major regions with variability compared to NC_018108.1 (Fig 2), and a possible additional region compared to NC_014914.1. (Fig 3) These regions contain a variety of genes in the annotation, and they can be defined by the conserved sequence flanking the variable regions. This makes it possible to identify each variable region by its upstream and downstream sequence, which are the same across all the isolates. The first two of these regions correspond with the first two of the three previously reported regions of difference (12), while the third previously reported region of difference appears associated with the small region visible on the BRIG alignment between 1,200,000 and 1,400,000.

Data obtained from SNP and coverage rates of the MiSeq reads from the 200 isolates support the variable regions observed from the BRIG output. In the 35,000 base pair contiguous windows, the percent coverage calculations show the greatest change in windows over the variable regions. The mean percent coverage across all 200 isolates is lowest and variance highest in the windows over the variable regions. (Fig 3) The calculated SNP rates did not consistently vary with the variable regions. While there does appear to be some level of correlation between the mean and variance of the SNP rates with the variable regions, it is less evident. (S3 Fig).

A pairwise alignment between each pair of genomes was performed to find the overall percent identity. A single, continuous alignment of the entire chromosome could not be achieved with Lastz, so a cumulative percent identity was calculated from the multiple segments of alignment and percent coverage of the genome was also calculated in order to clarify what percent of each isolate contains the stated level of identity. Breaks were commonly seen in the aforementioned variable regions. Overall identity in all cases is over 99%. However, there is a range in percent coverage that drops to as low as 86.56% for 79–1587 when compared with isolate 89–0490. The alignment between isolates 89–0490 and 98–0554 yielded a percent coverage of 89–0490 of 100.44% due to overlapping regions. The remainder of the pairwise comparisons yielded similar results. (Table 3).

**Table 3:** Percent identity and coverage calculated from Lastz output performing pairwise comparisons.

Isolates^a^	Identity^b^	Coverage^c^				Variable Regions Causing Breaks in the Alignment^d^
79–1587 vs 89–0490	99.54%	89–0490	91.19%	79–1587	86.56%	1,3,4
79–1587 vs 92–0972	99.34%	92–0972	97.12%	79–1587	93.11%	1,2,3,4
79–1587 vs 98–0554	99.57%	98–0554	90.67%	79–1587	88.26%	1,3,4
79–1587 vs 09–0932	99.49%	09–0932	98.73%	79–1587	92.85%	1,3,4
89–0490 vs 09–0932	99.36%	09–0932	99.72%	89–0490	98.76%	3,4
89–0490 vs 92–0972	99.46%	92–0972	99.62%	89–0490	97.82%	1, 2
98–0554 vs 89–0490	99.54%	98–0554	97.91%	89–0490	100.44%	1
98–0554 vs 09–0932	99.38%	09–0932	99.57%	98–0554	96.14%	3,4
98–0554 vs 92–0972	99.57%	92–0972	96.07%	98–0554	94.61%	1,2,3
92–0972 vs 09–0932	99.36%	09–0932	99.15%	92–0972	97.29%	1,2,3
79–1587 vs NC_018108.1	99.97%	NC_018108.1	97.99%	79–1587	97.58%	None
NC_014914.1 vs NC_018108.1	99.61%	NC_014914.1	98.08%	NC_018108.1	96.11%	1,2
09–0932 vs NC_014914.1	99.55%	09–0932	97.80%	NC_014914.1	94.27%	2,3,4

^a^Isolates aligned using Lastz.

^b^Percent identity of the sequence aligned between the two isolates.

^c^Percent coverage of the alignment for each genome.

^d^Variable regions causing breaks in the alignment are noted.

### Variable regions

Region 1 in Fig 2 is approximately 33,000 base pairs long and begins in the hypothetical protein that follows the first TonB-dependent receptor protein in the genome. The immediate upstream sequence is 5’- AAAGAAATGGGATTCACGGAGTCAAATAGT-3’, but this sequence is not unique in isolate 79–1587 and is highly similar to sequence throughout region 1 in NC_018108.1. The first unique upstream sequence occurs earlier in the initial hypothetical protein (5’- AAATTATATTATTGTTAATGACATTTGTTC -3’). The sequence immediately downstream is 5’- CGTGAATGTTTTAATTTCTCGAAAAAAGGA-3’. In isolate NC_018108.1 much of this section of the genome is annotated as repeat regions. Several repetitive segments are also present and supported by both the long read and short read data in isolate 79–1587. This area in all the genomes is composed almost entirely of hypothetical proteins. There are a total of 159 proteins identified in this region among the 7 isolates, and of those, only 23 proteins are not hypothetical and they are made up of only 4 annotated genes and two additional conserved domains. There are 2 Type VI secretion proteins, including *VgrG* and an ATP-binding protein identified in all isolates, as well as conserved domains DUF4150 and DUF3540 in NC_014914.1. While the function of both conserved domains is unknown, DUF4150 is part of the proline-alanine-alanine-arginine repeat superfamily that is associated with the *VgrG* protein. In addition to these Type VI secretion system proteins there is a pentapeptide repeat protein in NC_014914.1. Although, the pentapeptide repeat protein was not found in all genomes, it was revealed using BLASTx that the pentapeptide_4 superfamily conserved domain is present in all isolates at the same relative position indicating these genes are likely homologous. This region is much less pronounced in Fig 3 due to the similarity of the region across isolates 89–0490, 92–0972, 09–0932, and NC_014914.1. However, the observed variability in Fig 2 is supported by the coverage and SNP rate data. The contiguous window data demonstrates there is a decrease in the mean percent coverage and an increase in the variance of the percent coverage against all five assemblies across window 4, where region one occurs, with the most drastic changes being seen in isolate 79–1587 (percent coverage– 35.044%, variance– 0.036). Although the variance may seem small, it is six times larger than the neighboring windows that range from 0.0050 to 0.0055. The increase in variance is due to a large number of samples having low coverage (35.84% and below), while a few (15) samples have high coverage (98.80% and above) and no samples fall in the middle of this range. The remainder of the isolates are less divergent and demonstrate more similar changes in coverage and variance at lower rates than isolate 79–1587. Isolate 92–0972 has the second biggest changes with 88.779% coverage and a variance of 0.007.

A clustered regularly interspaced short palindromic repeat (CRISPR)/CRISPR-associated system (cas) region was identified in each isolate, and it was the main source of variation seen in region 2. This region starts in the center of the U32 family peptidase which is followed by the Cas3 endonuclease and ends just short of the oligopeptide ABC transporter substrate-binding protein *OppA*. It is marked by the sequence 5’-GGTGCGGAGAGATTTGGATTTGGTGGGGGT-3’ directly upstream and 5’-ATCTCTCCCACAAATTAGCCATTTCAAAGC-3’ immediately downstream and is approximately 15,000 base pairs in length. Four of the isolates, along with the NC_018108.1, have a Type I-C cas and identical repeat region in the CRISPR. The remaining isolate, 92–0972, has a Type I-F cas and a repeat region in the CRISPR that differs from the other isolates, but occurs in the same genomic region. This disproportionate occurrence of the different types of CRISPRs and cas is supported by the coverage findings where isolate 92–0972 shows a lower mean percent coverage (79.577%) than the other four isolates in window 7 where this region primarily occurs, and a greater variance than most other isolates (0.021).

Region 3 is approximately 20,000 base pairs in length and begins in a hemagglutinin protein with the sequence 5’-TACTACTAAATTAACTGATCTTACAAACGG-3’ and ends at the aminoacyl-tRNA hydrolase with the sequence 5’-ATGAAYACAYCCCTAAAACTTATCGTCGGT-3’. This region is a series of hemagglutinin and hypothetical proteins. Isolates 09–0932, 92–0972, and 89–0490 all have three hemagglutinin proteins identified, while the three remaining isolates have hemagglutinin and hypothetical proteins identified in their annotations. The hemagglutinin proteins do not all align well, however, using BLASTx it was apparent that all proteins (even those labeled as hypothetical) contain areas homologous to the conserved domains *LbR*_*YadA-*like (cd12820), a virulence factor, followed by *Hia* (COG5295), an autotransporter adhesin, which is also part of the *YadA*_anchor superfamily (pfam03895) as these domains are defined in the Conserved Domain Database (CDD) [31]. There are three other adhesins annotated in each genome. All three are more conserved, but contain areas homologous to *YadA*_anchor and *Hia* conserved domains as seen in the proteins in this region. In the coverage data isolate 79–1587 shows the largest decrease in percent coverage at 59.445% as well as the greatest variance at 0.028 in window 10, where variable region 3 occurs. Interestingly this region also shows a spike in the SNP rates of all the isolates, particularly in 09–0932 where the SNP rate goes from less than 0.002 in window 9 to 0.007 in window 10, then quickly returns to a rate of less than 0.002 in window 11. Isolates 92–0972, 98–0554, and 89–0490 also show a spike in their SNP rates in window 10 of approximately double the rates seen in the window nine. The variance of the SNP rates is increasing among all isolates in this window compared to the window previous.

Region 4 of Fig 1 begins in the unannotated region prior to conjugal transfer protein *TraL* (5’-ATGTAATGGGGATTAGAAATTACTGAAAAA-3’) and ends at the tRNA-*His* (5’-ATGGGGTGGCTGATGGGGCTCGAACCCACG-3’). It is approximately 44,000 base pairs in length, and only occurs in isolates NC_018108.1, 79–1587, and 98–0554. It contains several protein sequences including hypothetical proteins as well as genes that correspond to a type IV secretion system (T4SS). These include *TraL*, *TraQ*, *VirB9*, *VirB11*, in addition to *relaxase*, integrase, protein kinase, and other conjugal transfer proteins. In these sequences, the CDD shows homology is present to *VirB1* (PRK13864), *VirB4* (COG3451), *VirD2* (PRK13863), and *TrbM* (PRK13893). This region is split across two windows in the coverage and SNP analysis. Interestingly, in the case of both isolates, despite the first window containing a larger portion of the variable region, the second window shows a much lower percent coverage. In Isolate 98–0554 mean percent coverage is 68.856% and 38.333% respectively, and in isolate 79–1587 it is 72.664% and 20.463% respectively. The same observation is also made for the increase in variance across the two windows, which is 0.017 and 0.063 respectively in 98–0554 and in 79–1587, 0.015 and 0.107.

A possible fifth variable region can be observed in Fig 2. There is one primary protein responsible for the visible gap in the Fig 2, Protein C in isolate NC_014914.1. This protein is not annotated in any of the other genomes, but occurs just before a region that shows some lower levels of variability among isolates according to the SNP rate and coverage data in windows 29 and 30. The relative proximity to the variability in other isolates may indicate that region has some instability.

There are a small number of regions found in the study isolates that are not found in NC_018108.1. These areas are spread throughout the genomes and are supported by the BRIG output, where small gaps are visible, as well as the percent coverage and SNP rate analysis, where small changes are visible in various areas across the genomes. According to the annotation these areas are consistently composed of hypothetical protein and type IV secretion protein *Rhs*. One of these regions occurs in 79–1587, and it accounts for nearly all the difference in the size compared and the only significant break in the Lastz alignment to NC_018108.1. It begins at position 980,838, (5’- CTGGTTCAACTCCAGTATCGCCTACCACTA-3’) immediately after the tRNA-*Val* and ends at position 988,045, (5’-TATCTCAAATTTTACCCATGCTAAACTTTT-3’) approximately 60 bases upstream of the sel1 repeat family protein. There are seven hypothetical proteins annotated in the region. The last six of these proteins show homology to other documented sequences in *T*. *equigenitalis* including WP_013522661.1 (hypothetical protein), WP_013522622.1 (Protein C), WP_044956243.1 (*MobC*), WP_013522663.1 (hypothetical protein), WP_013522664.1 (*AlpA* family phage regulatory protein), and WP_013522665.1 (hypothetical protein). These proteins could not be located in NC_018108.1, and they are not replicated in 79–1587.

### Other regions of interest

A full set of *Sec* and *Tat* pathway genes were identified in all five genomes, along with several Type VI secretion system proteins occurring throughout the annotations[32]. Hauser et al. (2012) also noted *Flp* pili conserved genes that potentially aid in adhesion of the bacteria to tissues during infection, several of which were annotated in these genomes as well. These include *TadB*, *TadC*, and *CpaB*. Also annotated in each genome, and not previously discussed, were two *Fic* proteins. Several *Rec* proteins were also identified including *RecA*, *RecO*, *and RecR*. Surprisingly, *RecF* could not be located. Also present was *UmuC* and *UmuD*, the precursors of DNA polymerase V.

### Genome stability

Conversely to the variable regions, much of the genome appears to be consistent between all the isolates. This observation is supported by the whole genome alignments (Lastz, BRIG, Mauve) as well as the percent coverage and SNP rate data, which give insight to the level of stability of some regions of the genome. In particular, these regions include windows one through three and fourteen through twenty-eight of the SNP rate and coverage data. All of these windows have a coverage of greater than 99% except in four of the eighty-five instances (5 isolates x 17 windows), while the variance of the coverage is 0.005 at its peak in these areas. SNP rates also remain low in these regions with a maximum observed rate of 0.002. There are several other areas of each of the genomes that display high coverage and low variance and/or low SNP rates, but they do not occur simultaneously across all five genomes.

**Fig 4:**
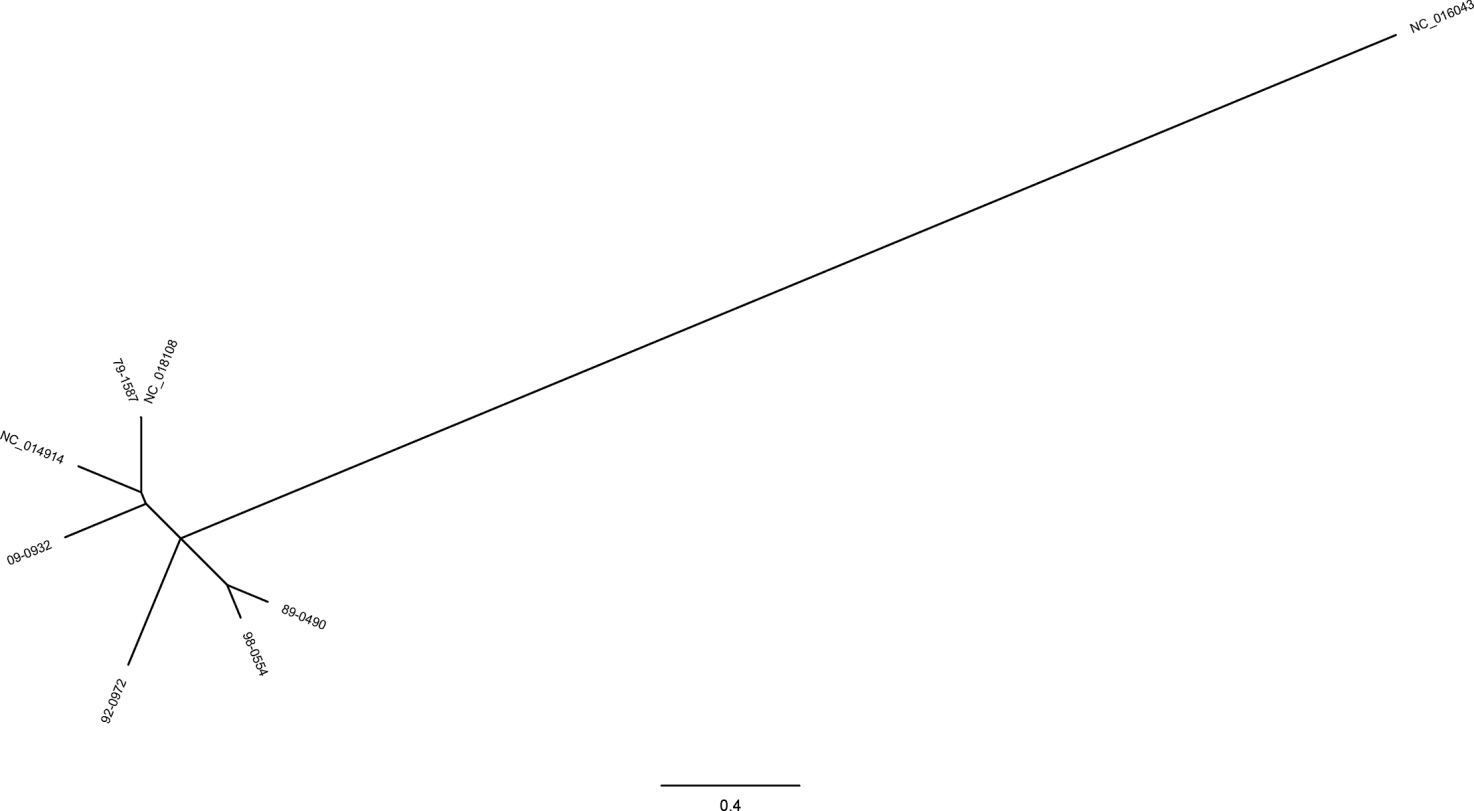
kSNP Maximum Likelihood (ML) tree for *T*. *equigenitalis*. The tree contains the six isolates discussed and NC_016043 (*T*. *asinigenitalis) as an out group*.

kSNP was used also used to evaluate SNP distance amongst study isolates and the references. A phylogenetic tree was built with kSNP based on the 5 study isolates, NC_018108.1, and NC_014914.1 in order to determine relative single nucleotide polymorphism (SNP) distance. (Fig 4) The method of determining SNPs in kSNP is based on the comparison of k-mers, which is not congruent with more standard reference based methods of determining SNPs [33]. Therefore, these numbers do not reflect absolute counts, but provide a relative number that is likely to be a lower bound of SNPs present between genomes. (Table 4) In this comparison the SNP distance between 79–1587 and NC_018108 is 41, a low number that is expected given the possible epidemiological link. The other isolates have a range of 1816–4295 SNPs, with a median of 3833 SNPs. Based on this median, the calculated SNP rate is 20 in every 10,000 base pairs.

**Table 4:** *T*. *equigenitalis* SNP distances from kSNP.

	09–0932	79–1587	89–0490	92–0972	98–0554	NC_014914	NC_016043	NC_018108
09–0932		3439	3922	4208	3855	3045	7817	3430
79–1587	3439		4140	4386	3924	2886	7836	44
89–0490	3922	4140		3896	1888	3949	7741	4132
92–0972	4208	4386	3896		3965	4269	7793	4381
98–0554	3855	3924	1888	3965		3730	7821	3919
NC_014914	3045	2886	3949	4269	3730		7861	2877
NC_016043	7817	7836	7741	7793	7821	7861		7834
NC_018108	3430	44	4132	4381	3919	2877	7834	

SNP distances were calculated in kSNP using all called SNPs with NC_016043 (*T*. *asinigenitalis*) as an outgroup.

### Antimicrobial resistance

A mutation previously shown to confer streptomycin resistance in other organisms is present in all three isolates that are known to be streptomycin resistant and is not present in the two isolates that are streptomycin susceptible in this study [34, 35].

## Discussion

*Taylorella equigenitalis* has caused disease in many countries throughout the world with a range in the severity of symptoms. The genomics of the species could provide valuable insight to the source of this variation, and better understanding of the genomic variation is also essential for more discriminatory genomic typing methods. This study provided a significant contribution to what is known about the genomic variability of the species as well as genetic features that may contribute to the virulence of the organism.

Region 1 shows significant heterogeneity among the isolates, but understanding possible explanations for this variation is difficult since the annotation is composed almost entirely of hypothetical proteins and thus the function of this region is unknown. In this region, isolate 79–1587 is clearly the most divergent isolate of the five studied, given the drastic decrease in coverage. The lack of a more substantial increase in SNPs throughout this region in any of the isolates can be partially explained by the decrease in coverage. Scaling the SNP rates based on coverage does yield higher SNP rates, but does not raise it above the rates in window 5 (except for 79–1587). The neighboring window five contains some variation in a portion of the autotransporter outer membrane beta-barrel domain-containing protein (visible in the BRIG alignment by the gap between regions two and three) which causes a large number of SNPs without loss of coverage. This may visually detract from the significance of the increase in the SNP rate in windows four, six, and seven.

In contrast to region 1, region 2 variation is expected. Development of the CRISPR over time is logical in the survival of the organism as it encounters viruses and bacteriophages, and it is only logical to expect isolates collected at different points in time from different geographical regions to have variation in the CRISPR. Both CRISPR types were also documented by Hauser et al. (2012), and upon further investigation, the repeat region in the CRISPR of 92–0972 is identical to that of the repeat in the CRISPR of isolate NC_021036.1, which was described by Hauser et al. in 2012 and also has a type I-F cas. BLAST results also show the repeat sequence is 1 SNP different than the repeat sequence found in the CRISPR of isolate NC_016043.1 (*Taylorella asinigenitalis*), also with a type I-F cas. Since this type of cas is known to be observed in other isolates, it is expected that the mean percent coverage would decrease in isolate 92–0972 due to the different cas. It is also expected that the variance in this region would be increased and is not unexpected that SNP rates didn’t raise substantially. It is highly likely that among the 200 isolates analyzed to generate this data, some of them will contain the same type I-F cas while many others will not. Although, the rate at which a type I-F cas is observed in this species is not currently known.

Region 3 and 4 variation is significant, as these regions contain genes that are associated with pathogenicity. While most of these isolates have not been studied for pathogenicity and the exact mechanism of pathogenesis has not be elucidated in *T*. *equigenitalis*, NC_018108 (NCTC 11184) was included in a study which demonstrated that this strain has both a high cellular invasion and intracellular replication rate compared to other strains of *T*. *equigenitalis* in that study [36]. Isolate 79–1587 has a possible epidemiological link to isolate NC_018108 and shows a great deal of genetic homogeneity according the annotation and Lastz results, suggesting it may also possess the same pathogenicity attributes of NC_018108. This isolate is again the most divergent isolate of the five study isolates in terms of mean coverage and variance of coverage in regions 3 and 4, although, it is closely followed by 98–0554 in region four. Region 4 is not identical in these two isolates, but there is a high degree of homology. Despite this similarity, in all phylogenetic trees presented here isolates 98–0554 and 79–1587 occur in separate clades with other isolates. They are not known to have originated from similar geographical regions and do not have any other known links, suggesting that region 4 was either acquired multiple times, or it was deleted from isolates at several points in time. Interestingly, the genes in region 4 are not insignificant, as they contain the only conjugal transfer proteins annotated in these isolates. It is unsurprising that the remaining three isolates show little to no variation in the same relative area of the genome as region four given that it appears to be completely excised from the isolates. Region 3 shows diversity amongst all five isolates and it is significant enough to cause a break in the pairwise alignment performed in Lastz in all but two comparisons (89–0490 aligned to 92–0972 and to 98–0554). Given the importance of the proteins in this region, it is possible there is a heavy selection pressure on these genes to increase survival of the organism.

Another possible factor in pathogenicity are the *Fic* proteins. Many *Fic* proteins are not well characterized, but of those that are many are known to be toxins secreted by Type III or IV secretion systems in bacteria [37].

To better assimilate the kSNP results, the same analysis was performed on 8 isolates representing each of the 8 documented lineages of *Mycobacterium tuberculosis complex* (MTBC), a group that is well studied and characterized genomically with an approximate genome size of 4.4 megabases [38, 39]. The tree and SNP matrix are shown in supplemental materials. (S4 Fig and S1 Table) A range of 1069–3451 SNPs, with a median of 2387 SNPs is observed in these samples. This yields is SNP rate of 5 in every 10,000 base pairs. The SNP distance results show that relatively, there is a great amount of genetic diversity among the isolates in this study compared to MTBC. With a genome size that is less than 40% of the size of MTBC, these isolates showed a median SNP count between epidemiologically unrelated isolates that is more than 50% greater than the median SNP count of the MTBC isolates in this comparison. The calculated overall SNP rate of 20 in every 10,000 base pairs with this method is consistent with the rates calculated for the contiguous windows. While much of the genome appears homologous, SNPs exist in nearly all proteins among these six isolates leading to a great deal of molecular diversity. One source of this diversity may be growth rate. Compared to MTBC, *T*. *equigenitalis* has a much faster growth rate as it can be grown under lab conditions in 24–48 hours compared to 7 days for fast growing *Mycobacterium* species and several weeks required for slow growing *Mycobacterium* species. [40, 41] Another source of this variation may be DNA polymerase V (*UmuC* and *UmuD*), which is annotated in all six genomes. This polymerase is a major component of SOS mutagenesis that is activated in response to DNA damage and makes a multitude of errors during DNA synthesis. One of these errors is base substitution which has been documented to occur at a frequency of approximately 10^−1^ to 10^−3^ [42].

Streptomycin resistance is the only documented antimicrobial resistance that varies between isolates of this species [3]. It has been reported previously in other organisms that streptomycin resistance is caused by a single point mutation in the *RpsL* gene (30S ribosomal protein S12) at the 43 residue. A base transition from A → G in the second position of the residue changes the lysine to arginine [34, 35].

This data provides greater molecular understanding of the pathogen, *T*. *equigenitalis*. Several methods were applied to evaluate the genome, and all were supportive of the same conclusions on regions of diversity. The identified regions of variability as well as regions of stability will be critical to advancing molecular typing methods. The diversity of these isolates as well as the completeness and quality of the assemblies adds to available data for future genomic studies on this organism.

## Supporting information

S1 Fig kSNP Maximum Likelihood (ML) tree of the NVSL diagnostic *T*. *equigenitalis*. This tree contains 200 diagnostic isolates from the NVSL repository. Clades are labeled with representative isolate that was sequenced with long read chemistry to achieve a complete genome.
(TIF)

S2 Fig kSNP ML tree of GenBank WGS *Taylorella* sp. isolates. A) *T*. *equigenitalis* isolates include 79–1587, 89–0490, 92–0972, 98–0554, 09–0932, NC_018108.1, NZZ_JRMO00000000.1, NC_021036.1, NZZ_LIYJ00000000.1, and NC_014914.1. B) *T*. *asinigenitalis* isolates includeHE681424.1 and NC_016043.1 C) An outgroup of *Bordetella pertussis* was used BX470248.1.
(EPS)

S3 Fig Mean and variance of SNP rate by window. Mean SNP rate and variance of the SNP rate of each isolate using the MiSeq reads from the 200 NVSL diagnostic isolates of *T*. *equigenitalis*. Results are given by 35,000 bp window.
(TIF)

S4 Fig kSNP ML tree of MTBC. This tree contains isolates from species *Mycobacterium tuberculosis* (CP005386.1, ERR159958, ERR234199, NC_002755.2, NC_018143.2, NC_021251.1), *M*. *bovis* (NC_002945.4), and *M*. *africanum* (NC_015758.1). These isolates represent major characterized lineages of MTBC world-wide.
(TIF)

S1 Table Additional sequence information. GenBank reference numbers and overall average depth of coverage.
(TIF)

S2 Table MTBC SNP distances from kSNP. SNP distances were calculated in kSNP using all called SNPs.
(TIF)
