# Supplementary figures and images for: Genomic diversity of Taylorella equigenitalis introduced into the United States from 1978 to 2012
Source: PLoS One. 2018 Mar 27;13(3):e0194253. doi: 10.1371/journal.pone.0194253 (PMC5870977; doi:10.1371/journal.pone.0194253)

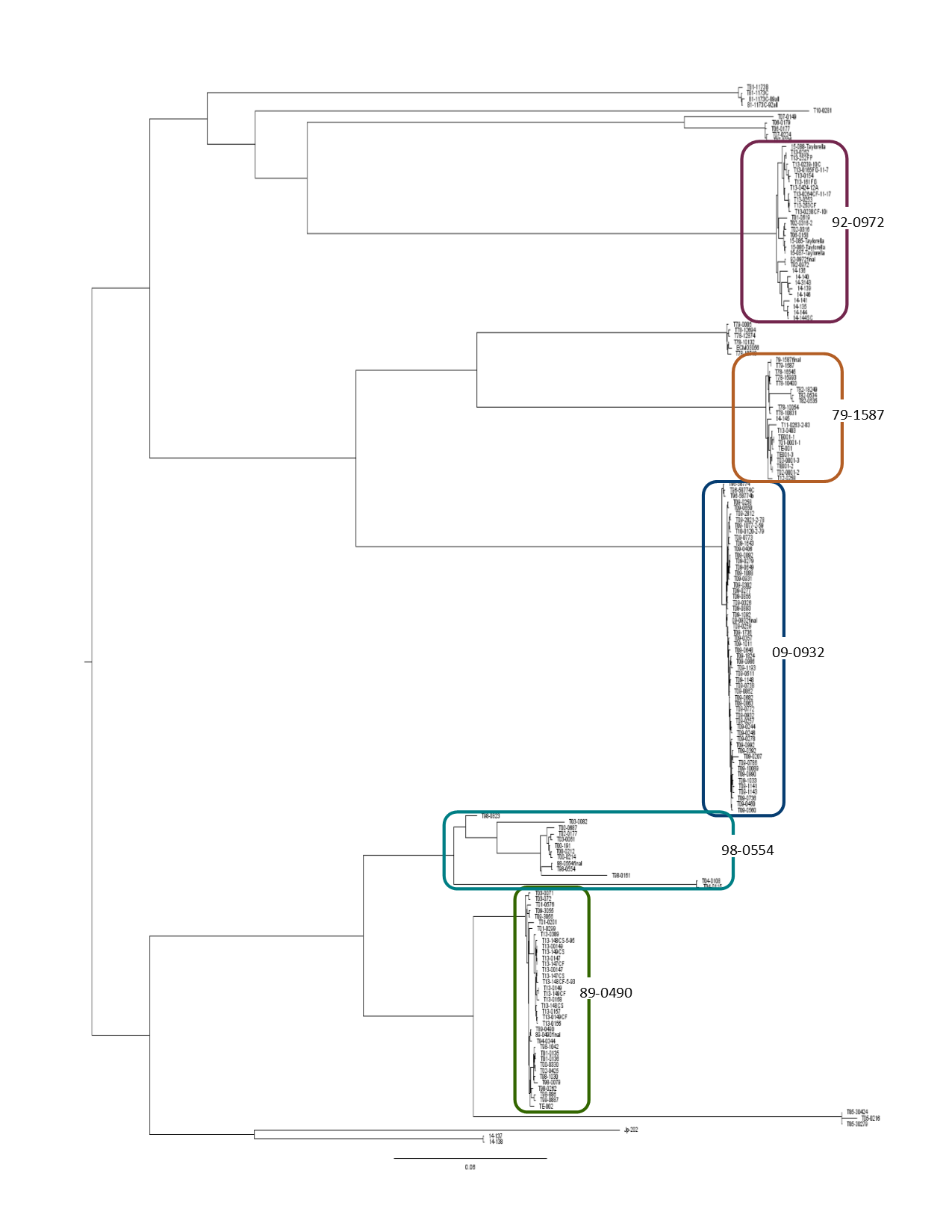

Supplement: S1 Fig — This tree contains 200 diagnostic isolates from the NVSL repository. Clades are labeled with representative isolate that was sequenced with long read chemistry to achieve a complete genome. (TIF) [file pone.0194253.s001.tif]

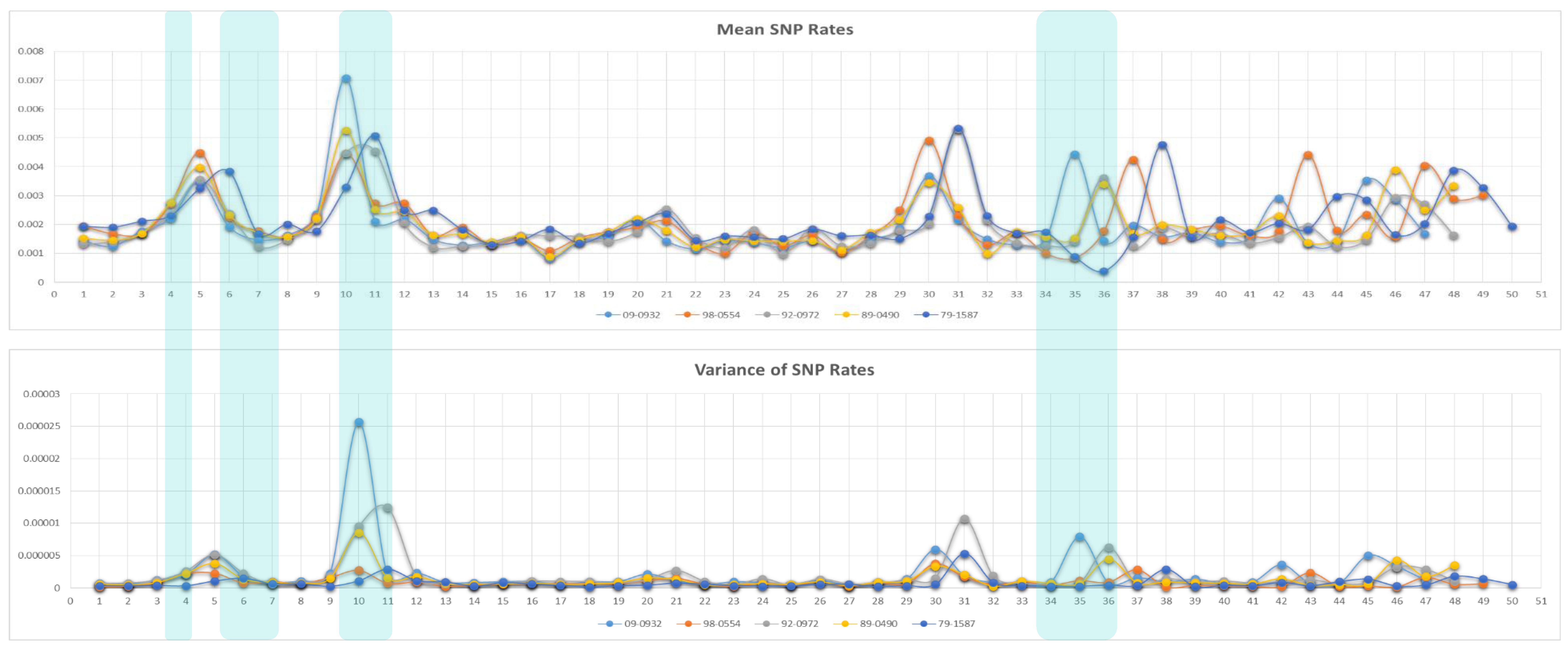

Supplement: S3 Fig — Mean SNP rate and variance of the SNP rate of each isolate using the MiSeq reads from the 200 NVSL diagnostic isolates of T. equigenitalis. Results are given by 35,000 bp window. (TIF) [file pone.0194253.s003.tif]
